# Doctors and Midwives

**Published:** 1896-08-22

**Authors:** 


### DOCTORS AND MIDWIVES.

THE discussion on the relations between medical men and midwives at the annual meeting of the British Medical Association was, on the whole, more worthy of the Association than some of the other debates in which the members have indulged this year. There would not really be any difficulty about the subject if the position of the midwives were once clearly defined and made sure by statute. In the meantime that position lends itself to certain varieties of underhand practice, and to a lowering of status as well as of remuneration among medical men. Midwives, in actual fact, work among the poor as irregular practitioners of one branch of the medical art. It follows from their defective training and other circumstances that they have no competency for dealing with any cases except those which are strictly normal. But in midwifery the abnormal is exceedingly frequent, the varieties of abnormality are many, and the dangers to poor women are often very great and urgent. From which also it follows that the midwife is very frequently under absolute compulsion to call in the doctor to help her out of her difficulties, or to lose the patient's life and take the consequences. Another circumstance connected with the undefined status of midwives as a class is the difficulty which the overworked doctor finds in utilising the services of the competent midwife for his own relief. The members of the British Medical Association considered whether it was justifiable for a medical man to help out a midwife in a case of difficulty, and also whether it was legitimate for a practitioner to employ a midwife as a midwifery assistant. Needless to say, all sorts of opinions prevailed upon the latter point; but about the former question, whether or not it was justifiable for a doctor to help out a midwife in a case of necessity, there was only one opinion. The suffering patient, everyone agreed, must be helped at once, and all matters of etiquette and questions of fee must be left for consideration until the endangered mother was brought to a condition of perfect and absolute safety. That was entirely as it should be. Humanity triumphed over etiquette and remuneration all along the line.

So far, good. But the fact still remains that the "undefined" midwife does a vast amount of harm. She creates a great deal of friction between doctors and doctors, as well as between herself and doctors; she fails to accomplish one half the good she might do both to patients and medical men; and all because she is not adequately trained and her status defined. For our part, we cannot see that it matters one straw whether the midwife be what is called "registered" or not registered, whether her cognomen be that of "midwife" or "midwifery nurse," or whether, indeed, she be called anything at all, so long as she is, first, adequately trained for her work, and, secondly, is allowed to work only under the supervision of a legally qualified medical man or medical woman. What is wanted before all things is to guard the safety of the patient; and the patient can only be adequately safeguarded by establishing, in every case, a chain of direct communication between her and a legally qualified medical man. The question ought not to be difficult; it is not really difficult in itself. It is made difficult because every individual medical man thinks his own scheme of dealing with it is the only possible one. As we so often insist here, the need of the poor is a small committee—very small—of thoroughly trusted medical men and lawyers, and that these shall thresh out a scheme, submit it for consideration to the whole profession, and incorporate any judicious amendments which individual critics may make; and then that the whole profession shall heartily accept the completed scheme and make it the basis of all rules regulating the relations between midwives and medical men. To this complexion things must assuredly come at last. The present *impasse* can no longer be tolerated by men of education and sense.

